# Major depression in outpatients attending a regional cancer centre: screening and unmet treatment needs

**DOI:** 10.1038/sj.bjc.6601578

**Published:** 2004-01-20

**Authors:** M Sharpe, V Strong, K Allen, R Rush, K Postma, A Tulloh, P Maguire, A House, A Ramirez, A Cull

**Affiliations:** 1 Division of Psychiatry, School of Molecular and Clinical Medicine, Kennedy Tower, Royal Edinburgh Hospital, Morningside Park, Edinburgh EH10 5HF, UK; 2 Cancer Research UK Psychological Medicine Research Group, Cancer Research Centre, Western General Hospital, Crewe Road, Edinburgh. EH4 2XR, UK; 3 Cancer Research UK, Edinburgh Oncology Unit, Western General Hospital, Crewe Road, Edinburgh EH4 2XR, UK; 4 Cancer Research Campaign Psychological Medicine Group, Christie Hospital NHS Trust, Stanley House, Wilmslow Road, Withington, Manchester M20 4BX, UK; 5 School of Medicine, University of Leeds, 15 Hyde Terrace, Leeds LS2 9LT, UK; 6 Department of Liaison Psychiatry and Cancer Research UK London Psychosocial Group, Adamson Centre for Mental Health, South Wing, St Thomas’ Hospital, London SE1 7EH, UK

**Keywords:** screening, major depressive disorder, usual care, antidepressants

## Abstract

A screening programme designed to identify cases of Major Depressive Disorder (MDD) in patients attending a Regional Cancer Centre outpatient department was established. It comprised two stages: (1) The Hospital Anxiety and Depression Scale (HADS) self-rating questionnaire administered by a touch-screen computer; (2) we interviewed patients with high scores on the HADS (15 or more total score) over the telephone using the depression section of the Structured Clinical Interview for DSMIV (SCID). A large consecutive sample (5613) of oncology clinic attenders was screened, and practical difficulties in the screening process were identified. The estimated prevalence of major depressive disorder (MDD) in the sample surveyed was approximately 8% (7.8%; 95% confidence intervals 6.9–8.5%). We assessed a consecutive series of 150 patients identified as having MDD to determine how many had received evidence-based treatment for MDD. Only half had discussed their low mood with their general practitioner, only one-third had been prescribed any antidepressant medication, and very few had taken a therapeutic dose for an adequate period. Very few had received psychological treatment or had been referred to mental health services. Most were receiving no potentially effective therapy.

Depression is an important and neglected problem in medical patients in general (Katon, 1996) and in cancer patients in particular (Chochinov, 2001). Previous studies have estimated the prevalence of Major Depressive Disorder (MDD) (American Psychiatric Association, 1994) in cancer patients to be as high as 50% (McDaniel *et al*, 1995). Major depressive disorder has a substantial impact on the quality of life of cancer patients and leads to reduced compliance with medical treatment and poorer outcome from it (McDaniel *et al*, 1995). Yet, studies show that psychiatric disorder goes unrecognised and untreated (McDonald *et al*, 1999; Fallowfield *et al*, 2001).

It has recently been recommended that all medical patients be screened for depression (Pignone *et al*, 2002). Screening can effectively detect cases of depression among oncology outpatients (Cull *et al*, 2001), many of whom would otherwise have been missed (Fallowfield *et al*, 2001). There is, however, little information on the performance of such screening systems ‘in the real world.’ How many cases of depression do such systems actually deliver and in how many of these is there evidence of an unmet need for treatment?

The study reported below is a description and evaluation of a two-stage screening programme for MDD implemented in a large cancer outpatient service in Edinburgh. The cases identified were subsequently entered into a trial of depression management, which is reported in a companion paper (Sharpe *et al*, 2003).

The aims of this study were: (1) to determine how many cases of MDD a clinically feasible two-stage screening process identifies among outpatients attending a large oncology outpatient service. (2) To identify the extent of unmet need for treatment of those patients identified as having MDD, who were potentially suitable for treatment in a cancer centre-based depression service.

## MATERIALS AND METHOD

### Sample

The study sample was a consecutive series of 5613 outpatients aged 18 years or over, with a diagnosis of cancer who attended selected outpatient clinics of a regional cancer service between September 1999 and September 2000. We chose clinics to screen with the aim of obtaining a sample with representation of both sexes, a wide range of ages and variety of cancer types. However, because we were interested in identifying those patients who would live sufficiently long to benefit from treatment given over several months, those clinics (such as those for lung cancer) in which many of the patients had a very short life expectancy were excluded. The clinics screened were for breast, gynae-oncology, bladder, prostate, testicular, colorectal, and mixed cancer types.

#### Description of attenders

I1n order to characterise the sample screened, the age, sex, and cancer diagnosis of 792 (14%) consecutive patients who attended the target clinics during 1 month in the middle of the sampling period (mid-June–mid-July 2000) had detailed clinical information abstracted from case notes.

### Identification of cases of major depression

In order to minimise the number of diagnostic interviews performed, patients with MDD were identified using a two-stage procedure. The first stage was intended to define a group likely to have MDD based on a self-rated questionnaire. Those scoring above the chosen threshold on this questionnaire and eligible to participate were then interviewed. Since it was difficult to arrange this interview during their clinic visit, it was conducted at home over the telephone.

#### Stage one

The Hospital Anxiety and Depression Scale (HADS) , a 14-item self-administered rating scale, was specifically developed to identify anxiety and depression in nonpsychiatric medical outpatients (Zigmond and Snaith, 1983). It excludes somatic symptoms of anxiety and depression. Two comprehensive reviews have found it to be a reliable and valid screening instrument (Herrman, 1997; Bjelland *et al*, 2002); it is quick and easy to administer and well accepted by patients. We used a total score cutoff of 15 because this was previously reported to offer good sensitivity and specificity (Ibbotson *et al*, 1994). We also examined the performance of the HAD by interviewing a consecutive series of 361 patients. This study (reported fully elsewhere) found that this cutoff performed adequately (sensitivity 87% and specificity 85%) and missed few interview positive cases. We administered the HADS by either a touch-screen computer (Cull *et al*, 2001) or when a computer was unavailable, by pen and paper. Good reliability between these two methods of administration has been previously reported (Velikova *et al*, 1999). We therefore considered the combined data collected by both these methods.

### Stage two

The section for diagnosing MDD from the Structured Clinical Interview for DSM-IV Axis I Disorders (SCID) (Spitzer *et al*, 1992) was administered over the telephone. This structured psychiatric interview ascertains the presence of the symptoms of MDD. If five or more symptoms from a list of symptoms, at least one of which must be depressed mood or anhedonia, have been present for 2 or more weeks, the diagnosis of MDD is made. In making the diagnosis of depression, all identified symptoms were counted without making a judgement about whether they should be attributed to cancer or to depression. This ‘inclusive approach’ is the most widely used and was chosen to maximise the sensitivity and inter-rater reliability of diagnoses (Koenig *et al*, 1997). All interviews were audio recorded for final blind ratings.

### Procedure

On arrival at the reception, patients were given an information sheet about the screening procedure and invited to complete the HADS questionnaire prior to their consultation. A research assistant was present in the clinic to offer help if required. Computer-generated HADS scores were produced from the touch-screen questionnaires and the paper form of HADS was scored by hand. These summaries were made available to the oncologist to review during the consultation. Every time the patient visited the oncology clinic during the trial period, screening was offered and records were kept in the patient notes of the HAD scores on each occasion.

Every patient with a HADS total score of 15 or over was contacted by telephone within 2 weeks of their clinic visit for the SCID interview (Ethical Committee approval for the study was contingent upon the oncologist giving permission for the patients to be contacted for interview). Three researchers who had undergone training in SCID interviewing conducted the interviews. The telephone-administered SCID has been previously shown to have good agreement with a face-to-face interview (Cacciola *et al*, 1999). Final ratings of MDD were made by consensus between the interviewer and a consultant psychiatrist (MS), who reviewed audio tape recordings of the interviews when necessary.

In all cases diagnosed as having MDD, the patient's general practitioner was notified of the diagnosis by letter. Eligible patients were asked for their consent to participate in a further research assessment.

### Patient characteristics and treatment needs

Our aim was to identify all those patients with MDD who could feasibly be managed outside specialist mental health services by oncology nurses and general practitioners. We therefore excluded from further assessment patients with the following characteristics: severe comorbid psychiatric illness (such as psychosis or bipolar disorder); severe cognitive impairment; chronic depression (i.e. depression present for a year or more prior to cancer diagnosis); depression associated with significant alcohol or substance abuse; and patients with complicating medical problems that required active management (such as cerebral metastases/poorly controlled epilepsy). As we were focusing on the needs of those with reasonable life expectancy, we also excluded those expected to die within 6 months.

Patients who met both diagnostic criteria for MDD and the above eligibility requirements were asked additional questions during the telephone interview. These aimed to ascertain: (a) when the current episode of depression started; (b) the number and duration of previous episodes; (c) what treatment, if any, the patient had received and was currently receiving; (d) current and previous use of mental health services; (e) current antidepressant drugs prescribed and the adequacy of dosage; and (f) adherence to the prescribed antidepressant drugs.

#### Definition of adequate treatment

There is uncertainty about what is the best treatment for MDD in patients with cancer. We have assumed that conventional evidence-based treatments for depression in the noncancer patients is applicable (Geddes and Butler, 2002), while accepting that further research is required to establish this. We defined the adequacy of dose of antidepressant drugs as that specified in the British National Formulary (BNF) (www.bnf.org). However, given recent evidence suggesting that the tricyclic antidepressant drugs may be effective at a lower dose of 75 mg (Furukawa *et al*, 2002), this was defined as the minimum effective dose for these agents. Psychological treatment was defined as a specified course of treatment with a recognised professional of at least two treatment sessions. Referral to Mental health services was defined to include referral to any discipline in the local NHS services.

### Analysis

First, the demographic characteristics of clinic attenders were described and supplemented with more detailed medical information on the 1 month subsample. Second, the numbers of patients included at each stage of the screening process were recorded and losses at each stage of the process accounted for. Third, the prevalence of MDD was calculated and an estimate was made that took into account patient attrition during the screening process. Finally, we described the characteristics and current treatment of patients who were both diagnosed with MDD and potentially eligible for management of their depression by an oncology department-based service.

### Ethical approval

The Local Research Ethics Committee approved the study.

## RESULTS

### Characteristics of the screened sample

We made a detailed case-note assessment, of 792 (14%) of the 5613 attenders screened. The majority (558 out of 792; 71%) were female with a mean age of 60 years. Breast cancer was the largest group (377 out of 792; 48%), with the remaining being approximately equally divided between prostate, bladder, testes, and colorectal and gynaecological cancers.

### Number of cases of MDD identified

Figure 1

**Figure 1:**
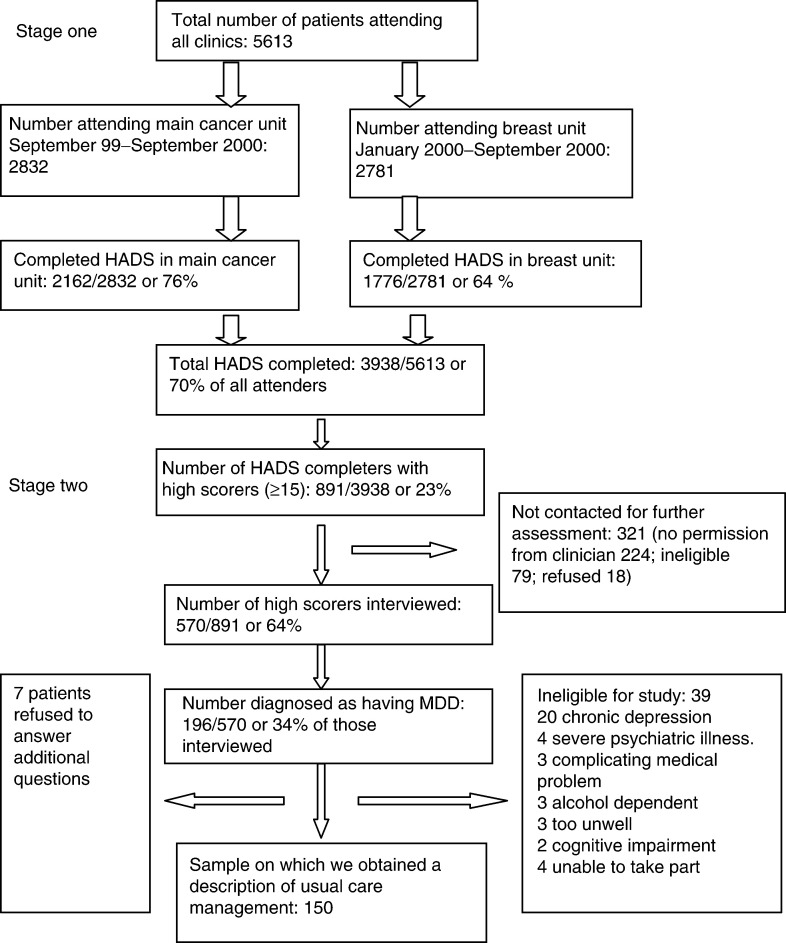
Number of patients included at each stage of the screening process.

is a flow chart showing the number of patients on whom data were recorded at each stage of the screening process. It includes both those screened in the general oncology clinics by a touch-screen computer, and those screened in the breast unit clinics by paper questionnaire. There was a higher completion rate of the HADS screening questionnaire in the oncology clinic (76% compared with 64%), yielding an overall completion rate of 70% (3938 out of 5613).

The data from these two outpatient departments were combined. In the combined sample, 891 out of 3938 or 23% (95% confidence intervals 21–24%) of completers scored above the cutoff of 15 or more on the HADS. Although the majority of these high scorers (570 out of 891; 64%) were assessed further by telephone interview, a substantial number of patients (321) were lost to further assessment at this stage. A number of patients (*n*=79) were ineligible for further assessment, almost all because of poor health or poor prognosis. For a further 224 patients, interviews were not achievable. The main reasons were failure to obtain permission from the oncologist to contact the patient, administrative reasons in 87 and refusal of permission in 27. Further losses were due to failure to achieve contact with the patient (*n*=96) and patient discharge from the clinic (*n*=14).

A comparison of the sample of patients on whom telephone interview was achieved with those on whom it was not (see Table 1

**Table 1:** Differences between those patients interviewed and those who were not interviewed

	**Telephoned**	**Not telephoned**		
**Variable**	**(*N*=570)**	**(*N*=321)**	**Statistic**	**Significance**
Mean (s.d.) age in years	61.6 (11.8)	58.6 (14.1)	*t*=−3.312	*P*=0.001
Percentage (number) female	89.1 (508)	70.1 (225)	*χ*^2^ 48.159	*P*<0.001
Mean (s.d.) total HAD score	19.2 (4.7)	19.6 (4.5)	*t*=1.57	*P*=0.117

) showed small but statistically significant differences in age and gender. The interviewed sample was slightly older and more likely to be that of a female. The mean total HADs score of the two groups was similar.

Major depressive disorder was diagnosed in 196 out of 570 (34%; 95% confidence interval 31–38%) of the high-scoring patients on whom SCID interviews were achieved. This represents 196 out of 891 (22%) of all the high scorers on the HADS if those not interviewed are included. In order to compensate for cases missed because of failure to obtain an interview and given that the mean HADs score of those interviewed was comparable to those not interviewed, we have assumed that a similar percentage of the high scorers not interviewed would have MDD. This is an additional 109 cases (34% of 321). We can then estimate the total number of cases of MDD in all high scorers, had we been able to interview them, as 305. This gives an overall estimated prevalence of MDD in those patients who completed the HADS screen of 305 out of 3938 (7.8%; 95% confidence intervals 6.9–8.6%). However, this estimated prevalence in the population of oncology attenders does not take into account those patients who refused to complete the HADS at initial contact (1675 out of 5613; 30%). Therefore, the ‘true’ prevalence rate may be higher than found in this sample, as there was an impression in the screening clinics that depressed individuals were more likely to refuse invitations to participate in the screening procedure.

### Description of patients with MDD

Of the 196 patients identified as having MDD, seven refused to provide further information. Of the remainder, 37 were considered inappropriate for treatment by a cancer centre depression service: 20 patients had chronic MDD (depression with onset at least 1 year prior to their first cancer diagnosis). Most of these patients had received or were receiving specialist psychiatric treatment. A further 19 patients were excluded for other reasons listed in Figure 1. The final sample of cases of MDD considered suitable for treatment in a cancer centre service and on whom assessments were competed was therefore 150. The characteristics of these 150 patients are shown in Table 2

**Table 2:** Demographic and medical characteristics of 150 patients with comorbid MDD.

*Demographic characteristics*	
Mean (s.d.) age in years:	57 (10.8) (range 31–83)
Female	135 (90)
*Cancer site*	
Breast	116 (77)
Bladder	4 (3)
Prostate	8 (5)
Testicular	3 (2)
Colorectal	7 (5)
Thyroid	1 (1)
Gynaecology	11 (7)
	
*Disease state*	
No active disease	117 (78)
Local disease present	17 (11)
Metastases	16 (11)
	
*Stage of treatment^a^*	
Active treatment	47 (31)
Monitoring only	103 (67)
Median (range) duration since first diagnosis in years	3 (0 to 29)

a Stage of treatment was operationalised as follows: on active treatment = patient undergoing chemotherapy or radiotherapy, under investigation or about to have surgery; monitoring = patient with no active disease but attending hospital for regular check-ups, monthly to annually in frequency (may be taking hormone treatment such as Tamoxifen). Number (percentage) with each characteristic unless otherwise specified.

.

The majority were females with breast cancer. Most had inactive disease, with only a minority currently undergoing cytotoxic treatment (other than taking anticancer medications such as tamoxifen) at the time of screening. The median interval since cancer diagnosis was 3 years, with a mode of 1 year. For a small number of patients, 10 or more years had elapsed since their original cancer diagnosis.

### Nature of MDD

The median duration of the patient's current depressive episode was 6 months. For half of the sample, this was their first episode of depression (see Table 3

**Table 3:** Depression characteristics of the study sample of 150 patients with comorbid MDD

*Median duration (range) of depressive episode (months)*	6 (1–216)
*Timing of onset relative to cancer diagnosis*	
With initial diagnosis	36 (24)
After diagnosis	97 (65)
With recurrence	17 (11)
	
*Previous depression*	
No previous episodes	77 (51)
One previous episode	49 (33)
Two or more previous episodes	24 (16)

Number (percentage) with each characteristic unless otherwise specified.

).

### Treatment received

**Figure 2:**
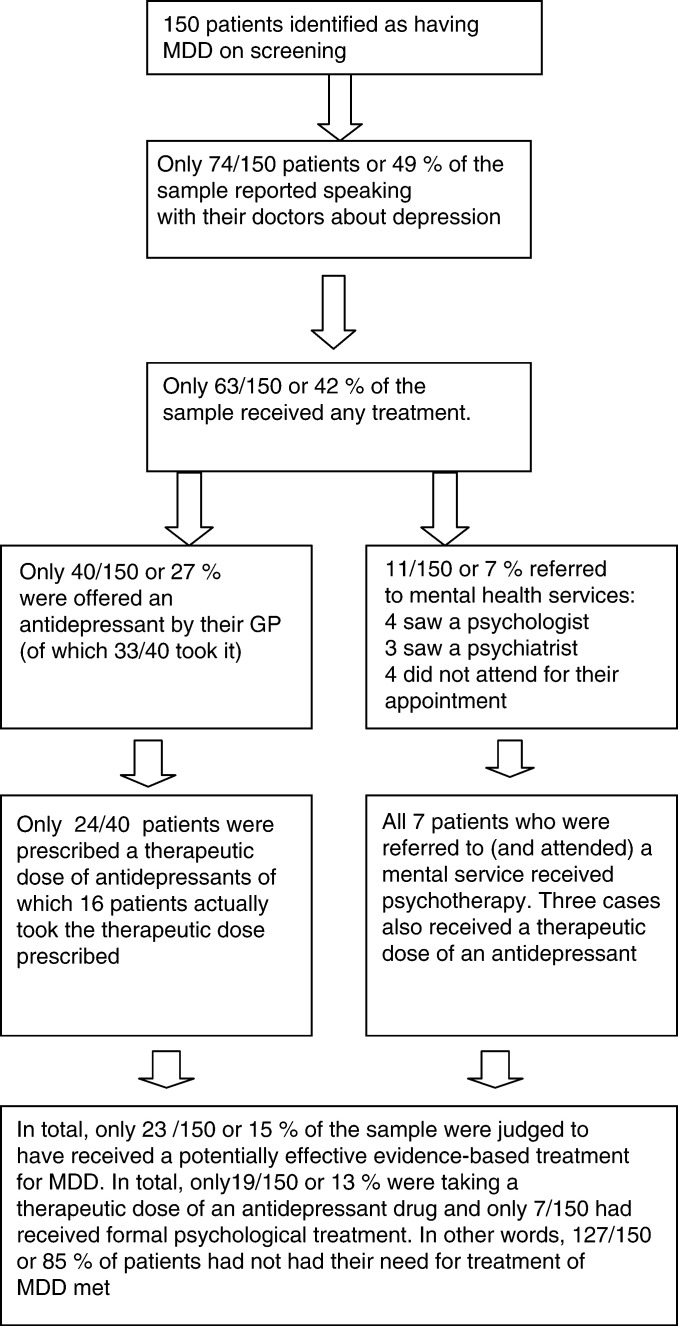
Flow chart documenting self-report of management of depression.

The treatment the patients with MDD reported having received for their current episode of depression is shown in Figure 2

. Overall, only 23 out of 150 (15%) of the patients were judged to have received or were currently receiving potentially effective evidence-based treatment for MDD. Likely reasons for the failure to deliver effective treatment were identified at every stage of their management and are shown in Figure 2.

First, only half (74 out of 150; 49%) of the patients said that they had even spoken with their GP or oncologist about feeling depressed. Second, less than a third (43 out of 150; 29%) reported being offered antidepressant drugs, and less than half of these were taking an adequate dose (as defined in methods) at the time of screening. Finally, only a small minority of patients (11 out of 150; 7%) had been referred to specialist mental health services (psychology or psychiatry) and only (7 out of 150; 5%) had received any formal psychological treatment for depression. These findings indicate a potentially substantial unmet treatment need.

## DISCUSSION

This study shows that it is possible routinely to identify cases of MDD in large numbers of oncology outpatients using a two-stage screening procedure. The proportion of attenders found to meet the criteria for MDD was lower than initially expected at approximately 8%. The most notable finding was that of the patients found both to have MDD and to be potentially suitable for treatment in a cancer centre-based depression service, the vast majority (85%) were not receiving any appropriate evidence-based treatment for depression.

### Screening programme

This particular screening programme aimed to identify patients who met diagnostic criteria for MDD as opposed to those with only more general distress. The HADs has been previously reported as being useful for this purpose (Bjelland *et al*, 2002), although others have reported it to be insensitive (Ramirez *et al*, 1995; Hall *et al*, 1999). A check of sensitivity in the population studied found it to be adequate at the cutoff used but that it required that three patients be interviewed for every case of MDD detected. The touch-screen administration system has also been previously found to be both generally acceptable to patients (Velikova *et al*, 1999) and to achieve a better completion rate than pencil and paper administration. The use of diagnostic interviewing over the telephone for high scorers on the HADS was also able to identify cases of MDD and was also, and perhaps surprisingly, very acceptable to patients (Allen *et al*, 2003).

The overall performance of the screening procedure was, however, impaired by ‘real-world’ practical and organisational difficulties. The main ones were: (1) an administrative failure to obtain HADs scores on all outpatient attendees, especially in the subsample who used pencil and paper questionnaires; (2) failure to achieve telephone interviews on all of those who scored highly on the HAD scale. Although these problems were at least partly remediable by changes in the screening protocol, they serve to remind us that the practical screening of large numbers of patients with limited resources is inevitably an imperfect process. The system was also opportunistic, screening patients only when they attended the oncology clinic. These times are not necessarily optimal for screening for depression. The effort invested in the screening to the number of cases identified was also relatively high, although the cost effectiveness of the process might be enhanced by using it to also identify patients with other problems such as anxiety, fatigue, and poor quality of life.

### Number of cases of depression identified

The observation that only 8 percent of the sample had MDD, even after correction for the omitted telephone interviews, was surprising. For example, a recent systematic review found a median prevalence of 15% among patients with advanced cancer (Hotopf *et al*, 2002). The relatively low prevalence in this study was probably because our sample was composed mainly of outpatients with inactive cancer, many of whom (particularly those in the breast service) were attending only for long-term follow-up, that is, most were not patients with advanced cancer and a few were undergoing active treatment for cancer. The sample characteristics reflected our choice of clinics and exclusion of patients with very limited life expectancy. It is, however, arguable that the group of patients we identified are precisely those suitable for treatment by an oncology service-based depression programme (Sharpe *et al*, 2003, companion paper). A different sample with a higher proportion of sick patients receiving active treatment would have been likely to have a higher prevalence of depression. Although our audit of the screening process suggested that the self-rated questionnaire missed few interview positive cases of MDD, there was a strong impression that some patients, especially men, either avoided the screening procedure or denied symptoms of depression that were in fact present. This phenomenon may also lead to underestimation of the true prevalence of MDD.

### Unmet treatment need

Importantly, only a minority of the cases of MDD identified appeared to have been explicitly recognised and only a small minority (15%) had received evidence-based therapy. A high level of unmet need for depression treatment has been recently reported (Fallowfield *et al*, 2001). Of more concern is the observation that similar findings were reported 20 years ago (Maguire *et al*, 1980). While it is possible that our estimate of unmet treatment needs was artificially increased because patients who had received effective treatment no longer meet the criteria for MDD, this finding has substantial implications for outpatient cancer services. Furthermore, the failings identified were at every stage of management; and include recognition, prescribing, patient adherence to medication, and availability of psychotherapy. They may also indicate patients’ own views and preferences about the need for a desirability of treatment for depression.

#### Recognition of depression

In our experience, the failure to recognise depression occurs in part because both doctor and patient are focused on the medical management of the patient's cancer. Even when detected, the depression may be disregarded as ‘understandable’ in someone with cancer. The implication is that questions about depression should be routinely asked and the responses acted on.

#### Prescribing of and adherence to antidepressant drugs

In patients in whom MDD had been recognised, two-thirds reported being offered an antidepressant drug, but only a small minority were actually prescribed a therapeutic dose. This is because a substantial proportion of these patients had decided not to take the drug and of those who had taken it, a further proportion had not achieved a therapeutic dose, probably because the patients’ general practitioner (George *et al*, 2000) did not increase doses. Even if a therapeutic dose was prescribed, a number either did not take it or had stopped taking it after only a short period. This poor adherence to antidepressant drug therapy has been previously noted (Lin *et al*, 1995). The implication is that prescribing is not enough – explanation and monitoring are also required.

#### Referral to specialist mental health services and receipt of psychotherapies

Very few patients reported having being referred to specialist mental health services and only five patients reported receiving an evidence-based psychotherapy such as cognitive behaviour therapy or interpersonal therapy (Geddes and Butler, 2002). These low rates probably reflect a combination of limited availability of local services, especially for patients seen as medically ill and stigma (Wessely, 1996). The implication is that integrated accessible, non-stigmatising, services are required.

#### Patients

In addition to failings in medical management, we also have to consider the extent to which cancer patients actually want to have their depression recognised and treated. In our experience, not all do. This issue is addressed further in a companion paper (Sharpe *et al*, 2003).

### Limitations

The main limitation of this study is that the sample reported on is not representative of all oncology outpatient attenders. This is because: (a) for practical reasons, only a selected number of clinics were screened, (b) as screening was designed to detect cases of MDD suitable for treatment in an Oncology Department Depression Service, patients with very limited life expectancy and patients with very chronic depression were excluded. It can, however, be argued that it is representative of those patients who are suitable for treatment by an oncology service-based depression treatment programme.

Despite our best efforts, there were a number of practical difficulties in the screening procedure and a large number of potential cases of major depression were lost during the process. Finally, while we have assumed that MDD in cancer patients would respond to treatments used for MDD in patients who do not have cancer, we acknowledge that the evidence supporting this assumption is limited and that further research into the effective treatment of depression in patients with medical conditions and comorbid depression is urgently required.

### Implications

These findings have implications for both practice and for further research. In clinical practice, the findings highlight the high rate of untreated depression in medical populations in general and cancer populations in particular. They also demonstrate the potential value of screening. There are, however, important caveats. First, there is, little point in screening unless it is associated with an intervention programme (Gilbody *et al*, 2001). We report on such a programme in a companion paper (Sharpe *et al*, 2003). Second, screening places a burden on both patients and staff. Further developments need to explore ways of making it more efficient and cost-effective. For example, it could be used to screen simultaneously for depression, anxiety, fatigue, and other symptoms as well as providing serial measures of quality of life for monitoring progress.

### Conclusions

It is possible to identify cases of MDD in patients attending a large and busy oncology outpatient department with only limited resources, especially if automated screening and telephone interviews are used. However, even with such a system, potential cases of major depression are likely to be missed. We suggest that such a system could be more cost effective if used to screen for a range of problems and not simply major depression.

The prevalence of major depression identified in ambulatory outpatients (most of whom were attending for follow-up rather than active treatment), with poor prognosis patients excluded, was surprisingly low. However, only a minority of patients who could potentially be treated for depression within the cancer service had actually received effective treatment. The failure to recognise and treat MDD in cancer patients deserves further attention. Specifically, we would argue that there is a need for the identification and treatment of depression to be better integrated with the patient's oncological care and to be carried out in collaboration with their general practitioner. We have piloted such a service and the results of this are reported elsewhere (Sharpe *et al*, 2003).

## FUNDING

The NHS Research and Development Programme for Cancer/Cancer Research UK and the Lothian University Hospital NHS Trust Cancer Research Fund funded this research.
